# Influences of Adenoid Hypertrophy on Children’s Maxillofacial Development

**DOI:** 10.3390/healthcare11212812

**Published:** 2023-10-24

**Authors:** Yulin Lan, Jieyi Chen, Shoucheng Chen, Yifan He, Fang Huang

**Affiliations:** 1 Hospital of Stomatology, Sun Yat-sen University, Guangzhou 510055, China; lanyulin@fsfy.com (Y.L.); chenjy679@mail.sysu.edu.cn (J.C.); chenshch8@mail.sysu.edu.cn (S.C.); 2 Guangdong Provincial Key Laboratory of Stomatology, Guangzhou 510080, China; 3 Foshan Maternity and Child Healthcare Hospital, Foshan 528000, China

**Keywords:** adenoid hypertrophy, maxillofacial development, MRI, airway, palate

## Abstract

This study aims to investigate the association between adenoid hypertrophy and facial development. A total of 388 children aged 1–13 years old who had undergone head MRI in Foshan Maternal and Child Health Hospital were collected, including 196 hypertrophic cases and 192 normal cases. The maxillofacial soft tissue indicators were measured and compared. The A/N ratio and adenoid thickness consistently increased with age in the hypertrophic group and the A/N ratio reached a maximum value three years earlier than the normal group. The pharyngeal airway space, vallecula of epiglottis to anterior plane distance of the third/fourth cervical vertebrae, angle of convexity, total angle of convexity, and the nasolabial angle in the hypertrophy group were smaller than those in the control group (*p* < 0.05). The thickness of adenoids, palate height, palate length, and tongue length in the hypertrophy group exceeded that of the control group (*p* < 0.05). To conclude, adenoid hypertrophy was associated with craniofacial features such as a convex facial profile, a narrowed nasopharyngeal airway, an elongated and heightened palate, a lengthened tongue or a lower tongue position. These findings emphasize the importance of early intervention for children with adenoid hypertrophy to mitigate potential adverse effects on maxillofacial development.

## 1. Introduction

Adenoid hypertrophy is an enlargement of the adenoid which is associated with mechanical obstruction and/or chronic inflammation in the nasopharynx [1]. Adenoid hypertrophy was prevalent with a prevalence rate of 49.70% from a total sample of 4778 children and adolescents, reported by a recent systematic review and meta-analysis [2]. Children under the age of six appear to be the most susceptible to adenoid hypertrophy because of the involvement of adenoid in the immune mechanisms at an early age. Typical symptoms of adenoid hypertrophy include obstruction of the upper airway, mouth breathing and sometimes dental malposition [1]. Adenoid hypertrophy will lead to long-term consequences if the condition is left untreated [3].

In accordance with the MOSS matrix theory, disruptions in respiratory function upset the equilibrium of perioral muscle forces, inducing alterations in mandibular position and culminating in malocclusion [4]. Prior investigations have posited that children engaging in mouth breathing typically manifest a facial profile with convex features, augmented lip thickness, and a subdued facial expression [5]. Additionally, these individuals exhibit posterior rotation of the mandible, heightened lower anterior face height, a constricted maxillary dental arch, as well as increased overjet and overbite of the anterior teeth [6,7]. The distinctive presentation of an anterior open bite characterizes this malocclusion as a Class II anomaly in alignment with Angle’s classification [8]. Nevertheless, certain studies challenge the notion that airway obstruction correlates with the anteroposterior positioning of the upper and lower jaw or the occlusal relationship within dental arches [9,10,11]. Some even posit that children who breathe through their mouths may display underdeveloped maxillary growth [12]. It is evident that the impact of mouth breathing on the craniofacial region’s development remains inconclusive in current research.

Previous research has predominantly employed X-ray cephalometric analysis to investigate the effects of adenoid hypertrophy on craniofacial development, but limitations in soft tissue resolution and positioning accuracy have been identified, potentially leading to underestimated adenoid sizes [13]. Recent studies have explored the use of MRI, a radiation-free imaging modality with superior soft tissue resolution, for assessing adenoid size and upper airway structures accurately and reliably [14]. Despite these advantages, no prior research has leveraged MRI to examine the influence of adenoid hypertrophy on craniofacial development.

This study employs MRI to measure and analyze craniofacial soft tissue parameters in children who have undergone MRI examinations. This study aims to investigate the association between adenoid hypertrophy and craniofacial development by comparing craniofacial soft tissue parameters between children with adenoid hypertrophy and those with normal adenoids. The results of this study may provide valuable insights for clinical interventions related to this condition.

## 2. Materials and Methods

This retrospective study obtained ethical approval from the Institutional Review Board of the Foshan Maternal and Child Health Hospital (FSFY-MEC-2020-066). All study subjects were informed their medical records would be used for research purposes, and parental consents were maintained. Before the commencement of the study, the examiner (Y.L.) was trained by an experienced orthodontist (Y.H.) and pediatric dentist (F.H.) with the intraclass correlation coefficient of 0.75.

### 2.1. Study Population

Initially, a cohort of 1750 children who underwent cranial MRI examinations at Foshan Maternal and Child Health Hospital between January 2014 and December 2022 was invited for this study, and their medical records were retrieved. According to the below inclusion and exclusion criteria, a final sample of 388 children was recruited. Sample size calculation, based on the formula N = μ_α_^2^P(1 − P)/δ^2^, with μ_α_ = 1.96, P = 34.46% [2], and δ = 0.05, yielded a required sample size of 347, which was met in this study.

**Figure 1:**
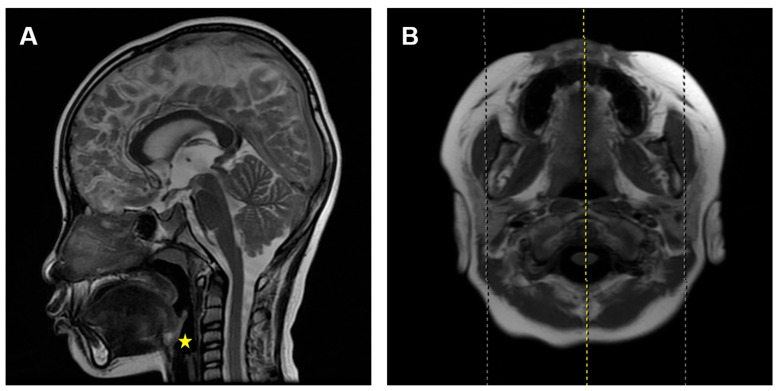
Mid-sagittal and cross-sectional head MRI images in T2W1 signal. (**A**) Mid-sagittal image. (**B**) Cross-sectional image. The yellow line represents the cross-sectional cutting position of the yellow star.

According to a previous study [15], the inclusion criteria were set as: (1) children who underwent MRI for reasons such as head trauma, febrile seizures, or suspected intracranial lesions, with no facial abnormalities other than adenoid hypertrophy, like nasal septum deviation, nasopharyngeal tumors, sinusitis, maxillary sinusitis, or delayed brain development; and (2) children with clear and complete mid-sagittal head MRI images in T2W1 signal (Figure 1).

### 2.2. Research Methods

**Figure 2:**
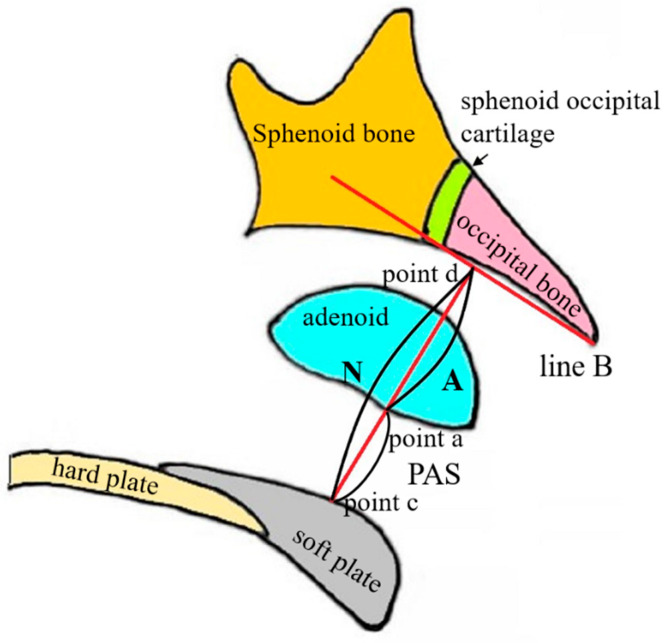
Schematic figure of adenoid measurements. Line B represents the line connecting the lower edge of the occipital slope to the posterior lower edge of the sphenoid vomer cartilage. Point a represents the most anterior point of the adenoid. Point d represents the intersection of a vertical line from point a to line B. Point c represents the intersection of the reverse extension line of ad and the upper edge of the posterior hard palate or the mid-anterior soft palate. A represents the distance between points a and d. N represents the distance between points c and d. Posterior Airway Space (PAS) represents the effective airway width between the soft palate surface and the adenoid surface, equal to the difference between lines N and A.

**Figure 3:**
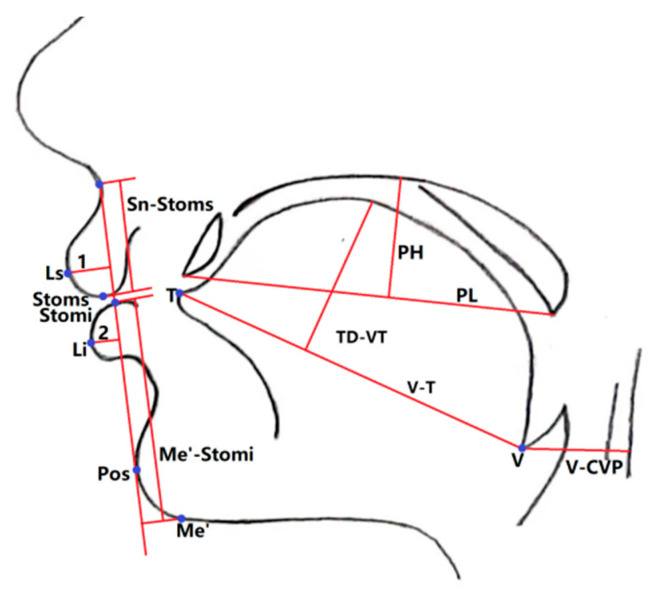
Schematic figure of distance measurements. Vallecula to the anterior plane of the third or fourth cervical vertebrae (V-VCP) represent the vertical distance from the vallecula point (V) to the plane of the third or fourth cervical vertebrae. Tongue Length (V-T) represents the line connecting the vallecula point (V) and tongue tip (T). Tongue Dorsum Height (TD-VT) represents the vertical distance from the highest point of the tongue dorsum contour to the V-T line. Palate Length (PL) represent the line connecting the anterior edge of the hard palate to the posterior edge of the soft palate. Palate Height (PH) represents the vertical distance from the highest point of the palate to the line connecting the anterior edge of the hard palate and the posterior edge of the soft palate. Upper Lip Length (Sn-Stoms) represents the vertical lines from subnasale point (Sn) and stomion point (Stoms) to the Sn-Pos line, with the distance between the two vertical lines. Lower Lip Length (Me’-Stomi) represents the vertical lines from soft tissue pogonion point (Me’) and stomion point (Stomi) to the Sn-Pos line, with the distance between the two vertical lines. Upper Lip Protrusion (1) represents the distance from the upper lip protrusion point (Ls) to the Sn-Pos line. Lower Lip Protrusion (2) represents the distance from the lower lip protrusion point (Li) to the Sn-Pos line.

**Figure 4:**
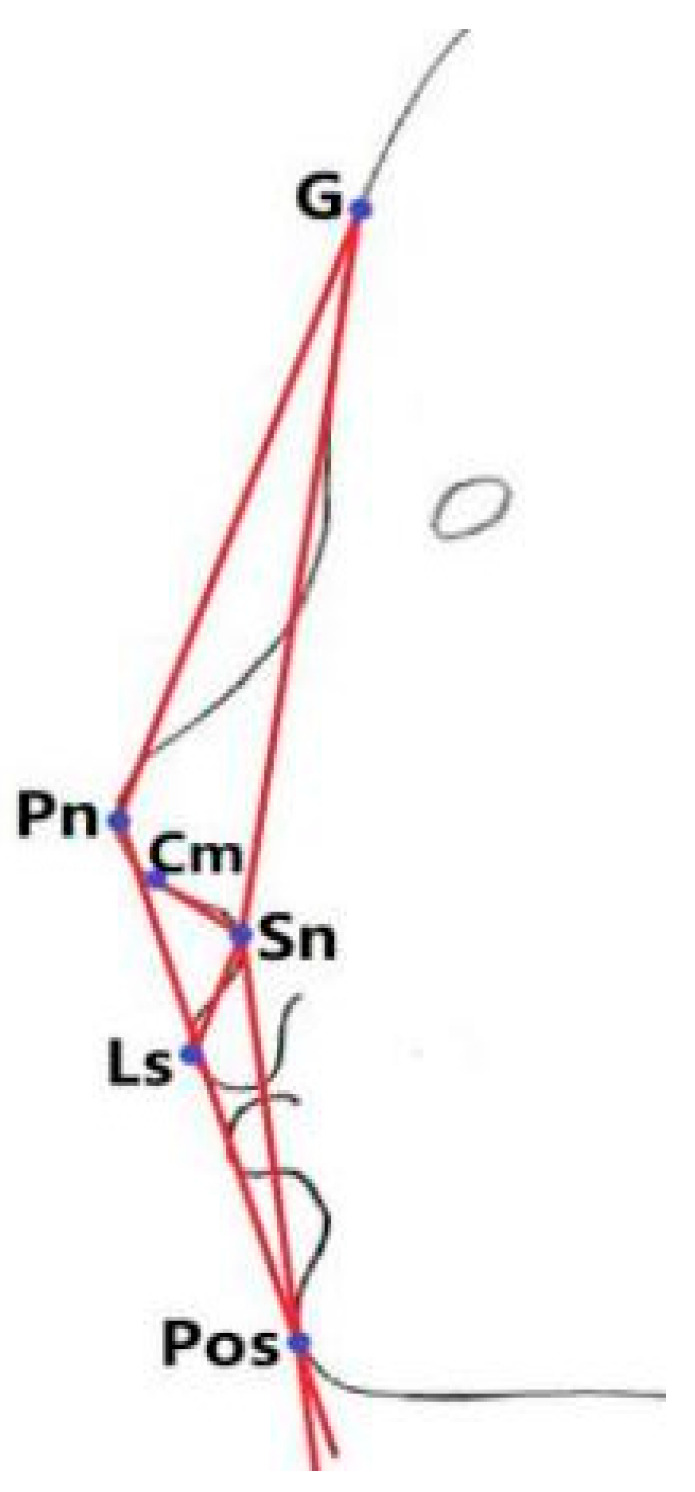
Schematic figure of angle measurements. Points: G (Glabella point), Cm (Columella point), Sn (Subnasale point), Pn (Nasion point), Ls (Labrale superius point), Pos (Soft tissue pogonion point). Total Facial Convex Angle (G-Pn-Pos). Nasolabial Angle (Cm-Sn-Ls). Facial Convex Angle (G-Sn-Pos).

The sagittal head MRI images were measured by one examiner utilizing the MRI examination system. Each measurement was measured twice and when the disparity was less than 1 mm or 1°, the mean value was adopted; otherwise, the measurement was reiterated. Each image was measured twice at an interval of one month between measurements, and the average of the two measurements was recorded. Ten percent of the MRI images was randomly selected for re-measurement contemporaneously, as well as after the wash-out period (one month), to assess the intra-examiner reliability. Adenoid measurements were conducted as shown in Figure 2 [16,17], while facial parameter measurements were conducted as shown in Figure 3 and Figure 4 [18,19]. Children who met the inclusion criteria were allocated into two groups based on the adenoid-to-nasopharynx (A/N) ratio [20]. Participants with an A/N ratio ≥ 0.71 were allocated to the hypertrophy group, and those with an A/N ratio ≤ 0.60 were normal group.

### 2.3. Statistical Analysis

The statistical analysis was executed using SPSS 21.0 software. Evaluation of age and gender differentials within the two groups was performed employing the chi-square test. Normal distribution and homogeneity of variance for facial parameters in both groups were assessed. When these assumptions were fulfilled, an independent sample *t*-test was employed; otherwise, a rank sum test was administered. *p* values < 0.05 were considered statistically significant. Inter- and intra-examiner agreements were assessed by calculating the intraclass correlation coefficient (ICC).

## 3. Results

### 3.1. Age and Gender Distribution of Study Participants

The hypertrophy group had 192 participants, comprising 123 males and 69 females, with an average age of 5.74 ± 3.13 years. The normal group consisted of 196 children, including 112 males and 84 females, with an average age of 5.86 ± 3.15 years. There were no statistically significant differences in age or gender between the two groups (*p* > 0.05), as outlined in Table 1 and Table 2. Intra-examiner agreements were excellent with ICC above 0.8 for both contemporaneous repetitions as well as repetitions at the one-month interval.

### 3.2. Changes in A/N Ratio and Adenoid Thickness with Age

**Figure 5:**
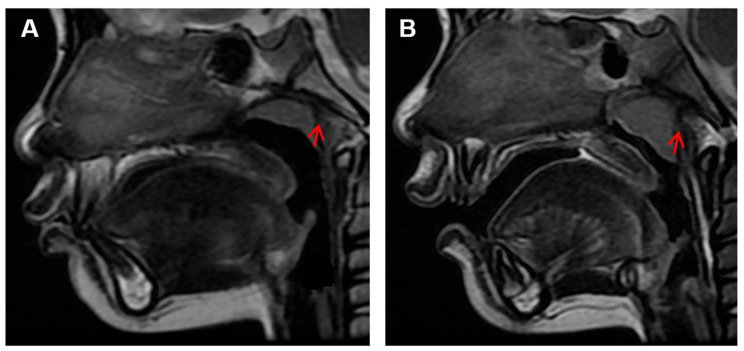
MRI Images of normal and hypertrophic adenoids. (**A**) The red arrow indicates a normal adenoid; (**B**) The red arrow indicates a hypertrophic adenoid.

Within the Normal group, the A/N ratio increased with advancing age up to 6 years, peaking at 0.56 by the age of 6. Within the Adenoid Hypertrophy group, the A/N ratio increased with advancing age until 3 years, culminating at 0.82 at the age of 3. Remarkably, the A/N ratio in the Adenoid Hypertrophy group markedly exceeded that in the Normal group across the age range of 1 to 12 years (Figure 5).

**Figure 6:**
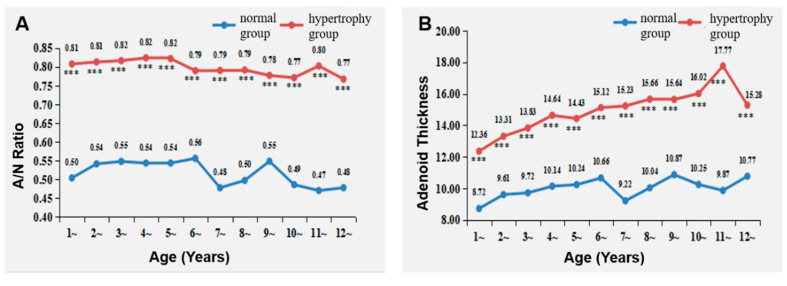
Changes in A/N Ratio and adenoid thickness with age. (**A**) Changes in A/N ratio with age; (**B**) changes in adenoid thickness with age. *** represents *p* < 0.001 for comparisons between hypertrophy and normal groups (*n* = 388).

Adenoid thickness in the Normal group displayed an elevation with age until the age of 6, peaking at 10.87 mm at 9 years. Comparatively, the Adenoid Hypertrophy group exhibited a continuous increase in adenoid thickness from ages 1 to 11, peaking at 17.77 mm. Notably, adenoid thickness in the Adenoid Hypertrophy group significantly surpassed that in the Normal group for ages 1 to 12 (Figure 6).

### 3.3. Comparison of Facial Profiles between the Two Groups

**Figure 7:**
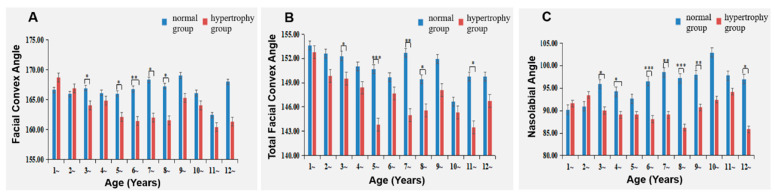
Impact of Adenoid Hypertrophy on Facial Profiles. (**A**) Facial convex angle; (**B**) total facial convex angle; (**C**) nasolabial angle. * represents *p* < 0.05, ** represents *p* < 0.01, and *** represents *p* < 0.001 for comparisons between hypertrophy and normal groups.

The Adenoid Hypertrophy group displayed a reduced facial convexity angle at ages 3, 5, 6, 7, and 8 compared to the Normal group. For all other ages, the differences did not attain statistical significance. The inter-group disparity progressively increased from ages 4 to 7 and subsequently decreased from ages 7 to 10 (Table 3 and Figure 7A).

Similarly, the total facial convexity angle was diminished in the Adenoid Hypertrophy group in comparison to the Normal group at ages 3, 5, 7, 8 and 11. Nonetheless, the differences were not statistically significant at other ages (Table 3 and Figure 7B).

Likewise, the nasolabial angle exhibited a reduction in the Adenoid Hypertrophy group compared to the Normal group at ages 3, 4, 6, 7, 8, 9 and 12. However, across all other age groups, the differences did not reach statistical significance (Table 3 and Figure 7C).

### 3.4. Comparison of Upper Airway Parameters between the Two Groups

**Figure 8:**
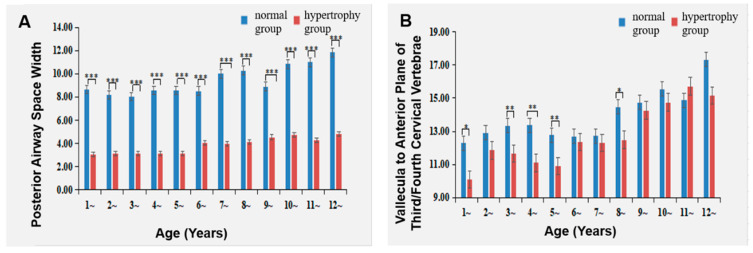
Impact of adenoid hypertrophy on upper airway. (**A**) Posterior airway space width (**B**) vallecula to anterior plane of third/fourth cervical vertebrae. * represents *p* < 0.05, ** represents *p* < 0.01, and *** represents *p* < 0.001 for comparisons between hypertrophy and normal groups.

The Adenoid Hypertrophy group displayed a narrower posterior airway space width compared to the Normal group at all ages. Within the Adenoid Hypertrophy group, the posterior airway space width measured less than 4 mm before the age of 6 and exceeded 4 mm after 6 years old (Table 4 and Figure 8A).

Furthermore, the distance from the uvula tip to the plane anterior to the third and fourth cervical vertebrae exhibited a reduction in the Adenoid Hypertrophy group compared to the Normal group at ages 1 3, 4, 5 and 8. However, for all other ages, the differences were not statistically significant (Table 4 and Figure 8B).

### 3.5. Comparison of Palate Height and Palate Length between the Two Groups

**Figure 9:**
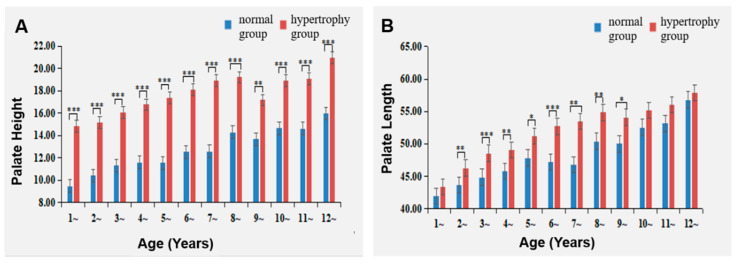
Impact of adenoid hypertrophy on palate height and palate length. (**A**) Palate height; (**B**) palate length. * represents *p* < 0.05, ** represents *p* < 0.01, and *** represents *p* < 0.001 for comparisons between hypertrophy and normal groups.

Palate height demonstrated an increase in the Adenoid Hypertrophy group compared to the Normal group at all ages. The disparity in palate height between the Adenoid Hypertrophy group and the Normal group gradually expanded between ages 3 and 6 (Table 4 and Figure 9A).

Moreover, palate length exhibited an increasement in the Adenoid Hypertrophy group compared to the Normal group at ages 2, 3, 4, 5, 6, 7, 8 and 9. Nevertheless, for all other age groups, the differences did not attain statistical significance. The disparity in palate length between the two groups gradually intensified between ages 4 and 7 (Table 4 and Figure 9B).

### 3.6. Comparison of Tongue Base Height and Tongue Length between the Two Groups

**Figure 10:**
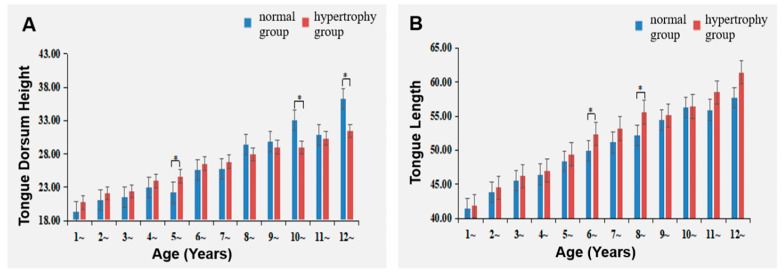
Impact of adenoid hypertrophy on tongue dorsum height and tongue length; (**A**) Tongue dorsum height; (**B**) tongue length. * represents *p* < 0.05 for comparisons between hypertrophy and normal groups.

Tongue base height was reduced in the Adenoid Hypertrophy group compared to the Normal group at ages 10 and 12. However, the tongue dorsum height exhibited an increase in the Adenoid Hypertrophy group at age 5. Conversely, tongue length was significantly increased in the Adenoid Hypertrophy group compared to the Normal group at ages 6 and 8 (Table 1, Table 2, Table 3 and Table 4 and Figure 10).

## 4. Discussion

The results of this retrospective case-control study demonstrated that adenoid hypertrophy primarily transpires between ages 2 and 8, inducing nasopharyngeal airway constriction, heightened facial convexity, and an elevated and elongated palate, persisting until age 12. Facial development encompasses two prominent growth phases, occurring at ages 5–10 and 10–15 [21]. This underscores the influence of adenoid hypertrophy on craniofacial development antecedent to peak growth periods, permeating both growth phases.

### 4.1. Changes in Adenoid Tissue with Age in Children

This research elucidated that within the Normal group, the A/N ratio attained its zenith of 0.56 at the age of 6, with adenoid thickness reaching its apogee at 10.87 mm by the age of 9. In contrast, the Adenoid Hypertrophy group witnessed the A/N ratio’s pinnacle at the age of 3, reaching 0.82, while adenoid thickness reached its zenith at 17.77 mm at the age of 11, marking a sustained escalation in adenoid size from ages 1 to 11. These results suggest that children with adenoid hypertrophy experience an earlier and prolonged phase of upper airway obstruction in the nasopharynx, compared to normal children. Moreover, adenoid hypertrophy-induced nasopharyngeal obstruction remains unabated until the age of 12. Songu M et al. [22] found that the adenoid thickness of children with sleep apnea increased continuously from 0 to 11 years old, which is similar to the results of this study. This corroborates previous findings that adenoid hypertrophy endures into adulthood, refuting the notion of age-related regression [23]. This underscores the imperative for early clinical intervention to mitigate prolonged nasopharyngeal obstruction.

### 4.2. Impact of Adenoid Hypertrophy on Facial Profile

The outcomes of this investigation disclose that, among participants aged 3 to 12 in the hypertrophy group, the facial convexity angle, total facial convexity angle, and nasolabial angle were smaller compared to those in the normal group. This implies that adenoid hypertrophy manifests as increased facial convexity as early as 3 years old. Parallel findings were observed by Cheng B et al. [23] and Souki BQ et al. [24], who noted a reduced nasolabial angle in mouth-breathing groups compared to control groups. However, in contrast to our study, they reported no significant difference in facial convexity angle between mouth-breathing and control groups. This discrepancy may be attributed to variations in study populations. Cheng B et al. focused on children with Class II skeletal patterns, aged 10 to 12 years, while Souki BQ et al. examined children with Angle Class I and Class II malocclusions. Unlike our study, these studies did not categorize subjects based on skeletal or occlusal relationships, potentially encompassing various types of malocclusions. Additionally, differences in body positions during MRI examinations (supine in our study versus natural standing in the aforementioned studies) may contribute to variations in facial prominence.

### 4.3. The Impact of Adenoid Hypertrophy on the Upper Airway

The outcomes of this study implied that the Adenoid Hypertrophy group manifested a narrower posterior airway space width and a diminished distance from the uvula tip to the plane anterior to the third and fourth cervical vertebrae, in comparison to the Normal group. Previous cephalometric analysis of 38 children aged 4 to 12 found that adenoid hypertrophy predominantly constricts the oropharynx and nasopharynx [25], concurring with our findings. Moideen et al. [26] established a significant correlation between upper airway obstruction symptoms and the A/N ratio; a larger A/N ratio signified more severe airway obstruction. The present study revealed that the Adenoid Hypertrophy group exhibited the highest A/N ratio values at ages 3 to 5, signifying pronounced airway obstruction within this age bracket. Moreover, the Adenoid Hypertrophy group displayed a reduced distance from the uvula tip to the plane anterior to the third and fourth cervical vertebrae predominantly at ages 3 to 5. These findings indicate that children within the Adenoid Hypertrophy group experienced airway obstruction with a width of less than 4 mm before the age of 6, which increased to greater than 4 mm thereafter. Lumeng et al. [27] observed that the peak incidence of pediatric obstructive sleep apnea-hypopnea syndrome (OSAHS) occurred between ages 2 to 5, aligning with the primary age range of airway obstruction witnessed in this study. This underscores the potential role of adenoid hypertrophy in exacerbating pediatric OSAHS development. Early intervention to alleviate nasopharyngeal obstruction attributable to adenoid hypertrophy, especially before the age of 6 [28], could contribute to the establishment of a healthy airway environment and avert pediatric OSAHS onset.

Adenoid hypertrophy exerts a discernible impact on the physical and mental well-being of children. Qi et al.’s study [29] revealed that adenoid hypertrophy correlated with a larger SNA angle and a smaller SNB angle, indicative of Class II malocclusion, a narrowed nasopharyngeal airway, compromised sleep quality, and stunted growth at aged 4 to 12. Macari et al. [30] examined 200 mouth-breathing children aged 1 to 12 via lateral cephalometric radiographs, concluding that children under 6 years old displayed more severe airway obstruction and pronounced maxillary protrusion. Our findings demonstrated that the Adenoid Hypertrophy group exhibited diminished facial convex angles, reduced total facial convex angles, and smaller nasolabial angles at the age of 3, underscoring the influence of adenoid hypertrophy on facial development as early as age 3, preceding previous reports. This variance may be attributed to the limited number of children under 3 years old in prior studies (less than 20 cases) and the challenges associated with conducting X-ray measurements and assessments on children within this age group, as postulated in our study. Early clinical intervention for adenoid hypertrophy can preempt craniofacial deformities, bolstering the physical and mental well-being of children.

### 4.4. The Impact of Adenoid Hypertrophy on the Palate

Our results unveiled that the dissimilarities in facial convex angles, palate height, and palate length between the Normal and Adenoid Hypertrophy groups progressively intensified with age, notably between ages 4 and 7. Furthermore, the study demonstrated that the Adenoid Hypertrophy group exhibited reduced palate height and palate length compared to the Normal group, with the effects of adenoid hypertrophy manifesting as early as age 1 for palate height and age 2 for palate length, enduring over time. Osiatuma et al.’s studied 90 children aged 3 to 12 with adenoid hypertrophy [7] revealed higher palatal heights in the premolar and molar regions across all age groups in the adenoid hypertrophy group compared to the normal group. Palatal length proved greater in the adenoid hypertrophy group aged 9 to 12 compared to the normal group, albeit within an older age range than this study. The divergence might be attributed to variations in measurement methods. It is noteworthy that previous studies did not encompass palate measurements in children below 3 years of age, possibly due to the challenges of acquiring X-ray and model measurements for this age cohort. Our study addresses a research gap pertaining to adenoid tissue and craniofacial development in young children, offering invaluable clinical insights. Notably, standard head MRI scans may not encompass the entire palate, rendering palate width measurements challenging. In clinical practice, when conducting MRI scans for adenoid assessment, it is advisable to ensure comprehensive palate coverage to facilitate palate width measurements.

### 4.5. The Impact of Adenoid Hypertrophy on the Tongue

This study unveiled that at ages 6 and 8, the Adenoid Hypertrophy group possessed a longer tongue length than the Normal group, while at ages 10 and 12, the Adenoid Hypertrophy group exhibited a shorter tongue base height. A long tongue may intensify stimulation in the sagittal direction of the palate, while a diminished tongue base height could lead to inadequate lateral palate stimulation, fostering elongation and narrowing of the palate. Additionally, at the age of 5, the Adenoid Hypertrophy group displayed a heightened tongue base height compared to the Normal group. This phenomenon might be attributed to young children employing tongue protrusion to facilitate airway opening in response to their respiratory demands. Elevated tongue base height could accommodate the elongation of the tongue due to continuous muscular stimulation and airflow during oral breathing, with the mandible adapting by posterior rotation to accommodate the elongated tongue. Subsequently, tongue base height may decrease. Importantly, MRI scans are conducted in the supine position, and alterations in posture may influence tongue length and tongue base height.

### 4.6. Limitations and Strengths of This Study

This study was a retrospective study, which lacks longitudinal comparisons. The study population was confined to children who underwent MRI examinations due to reasons such as head trauma, febrile seizures, and suspected intracranial lesions. This limited scope raises the possibility of systemic diseases affecting craniofacial development, introducing potential bias to the study results. It is crucial to acknowledge that the findings may be influenced by factors beyond adenoid hypertrophy. To address this limitation, future research should incorporate longitudinal studies to complement and validate the current results.

Nasopharyngeal endoscopy is the gold standard for diagnosing adenoid hypertrophy, and lateral cephalometric radiograph is commonly used to assess facial parameter measurements. This study adopted MRI measurements of adenoids, as well as facial development parameters, with less invasive operation, radiation, time-consumption, and child cooperation but better soft tissue resolution for assessment. MRI may serve as a decision-making compass for early adenoid hypertrophy intervention in younger children.

## 5. Conclusions

Adenoid hypertrophy was associated with craniofacial features such as a convex facial profile, a narrowed nasopharyngeal airway, an elongated and heightened palate, a lengthened tongue or a lower tongue position. These findings emphasize the importance of early intervention for children with adenoid hypertrophy to mitigate potential adverse effects on maxillofacial development.

## Figures and Tables

**Table 1:** Age Distribution of Normal and Hypertrophy Groups.

Age Distribution (Year-Old)	1~	2~	3~	4~	5~	6~	7~	8~	9~	10~	11~	12~
Normal Group	9	25	22	22	15	30	12	18	10	11	12	10
Hypertrophy Group	10	24	24	22	15	27	12	18	10	10	11	9

No statistically significant differences were observed between the normal and hypertrophy groups (*p* > 0.05).

**Table 2:** Gender Distribution of Normal and Hypertrophy Groups.

Group	Normal Group	Hypertrophy Group	Total
Male	112	123	245
Female	84	69	153
Total	196	192	388

No statistically significant differences were observed between the normal and hypertrophy groups (*p* > 0.05).

**Table 3:** Comparison of Facial Profiles of Normal and Hypertrophy Groups.

Angle Measurements	$\overset{-}{\chi }$ ± S		t Value	*p* Value
	Normal Group	Hypertrophy Group		
Facial Convex Angle (°)	166.56 ± 4.78	163.61 ± 6.68	5.01	<0.001 ***
Total Facial Convex Angle (°)	150.87 ± 5.31	147.48 ± 5.68	6.09	<0.001 ***
Nasolabial Angle (°)	95.63 ± 8.28	89.87 ± 7.97	6.98	<0.001 ***

*** represents *p* < 0.001 for comparisons between hypertrophy and normal groups (*n* = 388).

**Table 4:** Impact of Adenoid Hypertrophy on Facial Soft Tissues ($\overset{-}{\chi }$ ± SD).

Distance Measurements	$\overset{-}{\chi }\pm \;S$		t Value	*p* Value
	Normal Group	Hypertrophy Group		
Posterior Airway Space Width (mm)	9.16 ± 1.86	3.69 ± 1.36	33.01	<0.001 ***
Vallecula to Anterior Plane of third/fourth Cervical Vertebrae (mm)	13.65 ± 2.50	12.39 ± 2.34	5.11	<0.001 ***
Palate Length (mm)	47.69 ± 5.05	51.24 ± 5.07	−6.89	<0.001 ***
Palate Height (mm)	12.46 ± 2.17	17.45 ± 2.26	−22.16	<0.001 ***
Tongue Length (mm)	49.38 ± 5.83	50.69 ± 6.74	−2.05	0.041 *
Tongue Dorsum Height (mm)	25.59 ± 5.15	25.56 ± 4.28	0.06	0.953
Upper Lip Length (mm)	17.30 ± 1.91	17.44 ± 1.90	−0.74	0.463
Lower Lip Length (mm)	35.21 ± 4.29	34.98 ± 4.22	0.53	0.595
Upper Lip Protrusion (mm)	7.55 ± 1.29	7.73 ± 1.31	−1.42	0.156
Lower Lip Protrusion (mm)	5.53 ± 1.58	5.54 ± 1.91	−0.08	0.936

* represents *p* < 0.05 and *** represents *p* < 0.001 for comparisons between hypertrophy and normal groups (*n* = 388).

## Data Availability

The datasets generated and/or analyzed during the current study are available from the corresponding author upon reasonable request.
